# Supplementary material for: Genotype networks in metabolic reaction spaces
Source: BMC Syst Biol. 2010 Mar 19;4:30. doi: 10.1186/1752-0509-4-30 (PMC2858107; doi:10.1186/1752-0509-4-30)
Supplement: Additional file 1 — Supplementary material containing Figures S1 to S14. [file 1752-0509-4-30-S1.DOC]

# Additional File 1: Supplementary Figures S1 to S14 of

# “Genotype networks in metabolic reaction spaces”

Areejit Samal, João F. Matias Rodrigues, Jürgen Jost, Olivier C. Martin, Andreas Wagner

**Figure S1: The space of viable genotypes gets rarefied with decreasing *n*.** The horizontal axis shows the number *n* of reactions in a genotype and the vertical axis shows on a linear scale the estimated fraction of random genotypes contained in the viable space *V(n)* for three different chemical environments, glucose, acetate and succinate, respectively. The black dashed curve shows the analytical prefactor as function of the number *n*.

**
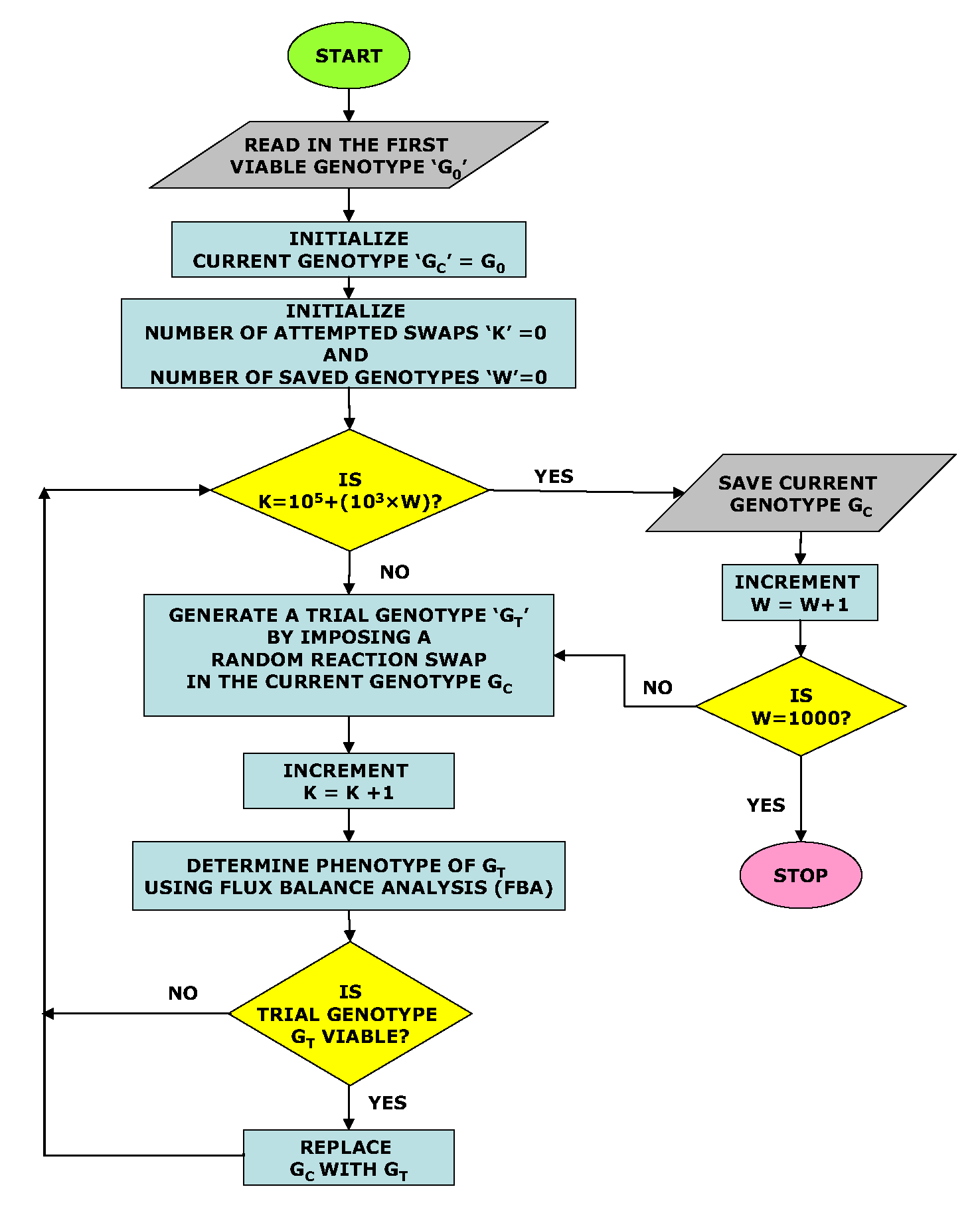
**

**Figure S2: Flowchart describing the logic of the MCMC algorithm.** To start the Markov chain, a first viable genotype is necessary. Beginning with the initial genotype, we perform 10^5^ Markov Chain steps to erase the memory of the initial genotype. After this initial phase, we continue the MCMC procedure to sample the genotype network and save every 1000^th^ genotype generated. We terminate the Markov chain after saving 1000 genotypes. Note that the length of the run (and the choice of saving frequency) should be long enough to obtain a meaningful sample of viable genotypes using this algorithm.

**Figure S3: Autocorrelation time of a typical MCMC run.** The horizontal axis shows the number of attempted swaps in an MCMC run and the vertical axis shows the average Hamming distance between the starting and current genotype for three environments (glucose, acetate and succinate). This distance grows and then saturates with a characteristic time scale τ of about two thousand attempted swaps for all three environments. Data are shown for *V(n)* with *n*=900.

**Figure S4: Acceptance rate of the MCMC procedure.** The horizontal axis shows the number *n* of reactions in a genotype and the vertical axis shows the acceptance rate *A* of the MCMC transition steps (reaction swaps). Results are shown for three different environments (glucose, succinate, and acetate). One sees *A*>0.2 for *n*=300 or more which is our regime of interest. Note that during the MCMC sampling of *V(n),* the super-essential reactions do not participate in the swaps.

**Figure S5: Narrow distribution of mutational robustness *R_μ_* for genotypes in *V(n).*** The horizontal axis shows the mutational robustness *R_μ_*, and the vertical axis shows the frequency of genotypes with the corresponding value of *R_μ_* in a random sample of 1000 viable genotypes in the glucose minimal environment with the viability constraint taken as having strictly positive biomass flux and the number *n*=700 reactions. We also display the normal distribution with sampled mean and theoretically predicted variance as a dashed black curve (see text for details). The figure shows that there is very little variation in *R_μ_* across random viable genotypes and the normal distribution agrees relatively well with the sampled distribution.

**Figure S6: Narrow distribution of mutational robustness *R_μ_* for genotypes in *V(n)* with modified biomass formula*.*** The modified biomass formula was constructed by starting from the reference one (that of *E. coli*) and randomly perturbing the stoichiometry of each biomass metabolite by up to 20%. The horizontal axis shows the mutational robustness *R_μ_* and the vertical axis shows the frequency of genotypes with the corresponding value of *R_μ_* in a random sample of 1000 genotypes viable in **a)** glucose, **b)** acetate and **c)** succinate environment with the viability constraint taken as having strictly positive biomass flux with the modified biomass formula. **d)** The distribution of mutational robustness *R_μ_*  in 1000 random viable genotypes for the glucose environment with viability constraint taken as biomass flux at least as large as the *in silico* *E. coli* biomass flux with the modified formula. In all cases, the number *n* of reactions is equal to that in *E. coli* (*n*=831). The figure confirms that *E. coli* has atypical mutational robustness even with the modified biomass formula.


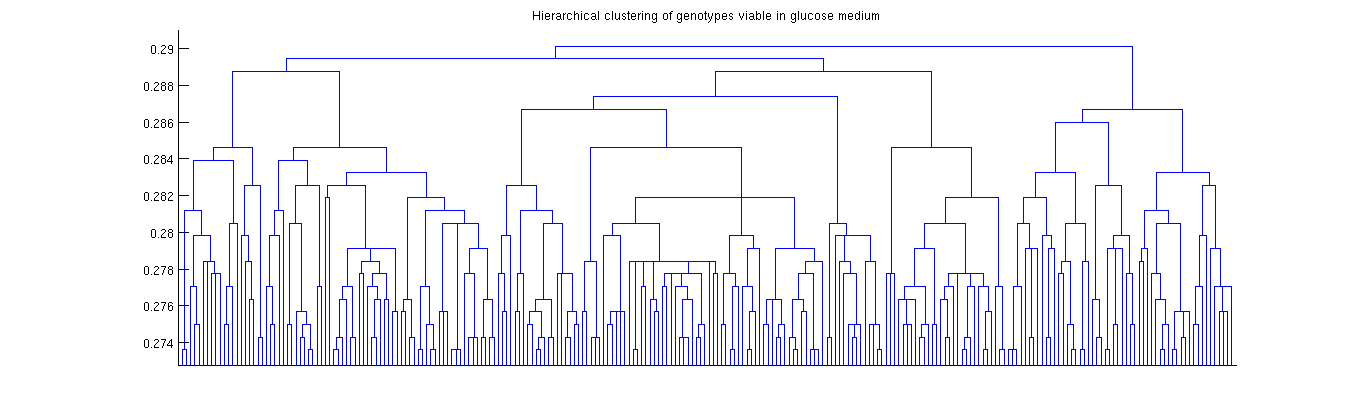


**Figure S7: Hierarchical clustering of genotypes in *V*(*n*).** The figure shows the dendrogram obtained by hierarchically clustering the 1000 sampled viable genotypes for glucose minimal environment with *n*=831 (the value for *E. coli*). Here, Hamming distance was used as the distance measure, followed by average linkage clustering as implemented in MATLAB 7.7. There is no evidence for multiple clusters.

**Figure S8: Principal component analysis of reactions occurring in random viable genotypes.** We have organized the 1000 sampled viable genotypes for glucose minimal environment with *n*=831 (the value for *E. coli*) into a matrix where each row is a bit string associated with one genotype. When we read this matrix one column at a time, we have a bit string of length 1000 that reflects the occurrence of each reaction in the global reaction set in our genotype sample. We subjected these bit strings for reaction occurrence to a principal component analysis. The horizontal axis shows the first principal component and the vertical axis shows the second principal component. We find that the first principal component correlates well with the rank of the reaction. To make the association between the first axis and reaction rank visible, we have colored the reactions according to their rank (red for ranks close to 1, indigo for ranks close to 2902). The data are clearly heterogeneous, resembling a comet with a dense head on the left and a spread-out tail on the right. The comet's head is formed mostly by blocked reactions, while the tail of the comet is enriched in essential reactions.

**Figure S9: Relative size of viable spaces for three different environments.** The horizontal axis shows the number of reactions in a genotype, and the vertical axis shows the relative size of the viable space for one environment relative to that for glucose. We here considered the viable spaces for three different environments, glucose, acetate and succinate, and found that the size of the viable space for glucose is greater than that of succinate, which is in turn is greater than that for acetate.

**Figure S10: Probability of a genotype to be viable in multiple environments.** The horizontal axis shows the number of reactions in a genotype and the vertical axis shows the probability that the genotype is simultaneously viable in several environments. We show the data for all three pairs and one triplet of the three environments (glucose, acetate and succinate). The probabilities shown are normalized by the fraction of genotypes that are viable in the glucose environment.

**Figure S11: Histogram of the number of additional environments in which random genotypes are viable.** The horizontal axis shows the number *E* of environments out of the 88 minimal environments in which a random genotype is viable (apart from the single environment where it was sampled from). The vertical axis shows, as a function of *E*, the corresponding number of genotypes found in our random sample of 1000 genotypes. The 1000 random viable genotypes were sampled to be viable in the **a)** glucose, **b)** acetate and **c)** succinate environment.

**Figure S12: Mutational robustness *R_μ_* increases with *n* for genotypes viable in multiple environments.** **a)** The horizontal axis shows the number *n* of reactions in a genotype, and the vertical axis shows the average mutational robustness of sampled genotypes that are simultaneously viable in two different environments as a function of *n.* The data are shown for sampled genotypes that are viable in all three possible pairwise environment combinations involving glucose, acetate and succinate. **b)** The horizontal axis shows the number *n* of reactions in a genotype and the vertical axis shows the average mutational robustness of sampled genotypes viable in all three environments (glucose, acetate and succinate) as a function of *n.*

**Figure S13: Narrow distribution of mutational robustness *R_μ_* for genotypes viable in multiple environments.** The horizontal axis shows the mutational robustness *R_μ_*, and the vertical axis shows the frequency of genotypes with the corresponding value of *R_μ_* in a random sample of 1000 genotypes that are viable **a)** in the glucose and succinate minimal environments; and **b)** in all three environments (glucose, acetate and succinate). The viability constraint is that of strictly positive biomass flux; the number *n*=831 of reactions is equal to that of *E. coli*. The figures show that there is very little variation in *R_μ_* across random viable genotypes and that the *E. coli* genotype is an outlier compared to random viable genotypes.

**Figure S14:** **Clustering of genotypes in *V(n)* for glucose, succinate and both environments**. The figure shows the first two principal components for the randomly sampled viable genotypes in the three considered chemical environments (glucose, succinate and both) with *n* equal to that of *E. coli*. The horizontal axis shows the first principal component, and the vertical axis shows the second principal component. The figure also shows the center of mass of each of the three sets as squares.
